# Current Therapies for Human Epidermal Growth Factor Receptor 2-Positive Metastatic Breast Cancer Patients

**DOI:** 10.3389/fonc.2018.00089

**Published:** 2018-04-03

**Authors:** Alexey A. Larionov

**Affiliations:** Department of Medical Genetics, School of Clinical Medicine, University of Cambridge, Cambridge, United Kingdom

**Keywords:** HER2, metastatic breast cancer, trastuzumab, pertuzumab, lapatinib, trastuzumab-emtansine, triple-positive breast cancer, therapies

## Abstract

The median survival of patients with human epidermal growth factor receptor 2 (HER2)-positive metastatic breast cancer (MBC) has more than doubled, since the discovery of HER2-targeted treatments: it rose from less than 2 years in 2001 (prior introduction of trastuzumab) to more than 4 years in 2017. The initial generation of HER2-targeted therapies included trastuzumab with taxanes in the first line, followed by the addition of lapatinib and by a switch to another cytotoxic agent after progression. Results of CLEOPATRA, EMILIA, and TH3RESA trials have changed this clinical practice. The current consensus includes horizontal dual blockade (trastuzumab + pertuzumab) with taxanes or vinorelbine in the first line, followed by trastuzumab-emtansine (T-DM1) in the second line, with addition of lapatinib in the later lines of treatment. However, the fast and simultaneous development of new drugs led to a relative shortage of clinical evidence to support this sequence. Triple-positive breast cancers (TPBC), which express both hormonal receptors and HER2, constitute nearly half of HER2-positive cases. For these tumors, the current consensus is to add endocrine therapy after completion of cytotoxic treatment. Again, this consensus is not fully evidence-based. In view of the recent progress in treatment of estrogen-receptor positive breast cancers, a series of trials is evaluating addition of CDK4/6 inhibitors, aromatase inhibitors or fulvestrant to HER2-targeted and cytotoxic chemotherapy in TPBC patients. Despite the remarkable progress in treatment of HER2-positive breast cancer, metastatic disease is still incurable in the majority of patients. A wide range of novel therapies are under development to prevent and overcome resistance to current HER2-targeted agents. This review discusses pivotal clinical trials that have shaped current clinical practices, the current consensus recommendations, and the new experimental treatments in metastatic HER2-positive breast cancer.

## Introduction

Human epidermal growth factor receptor 2 (HER2) was discovered as a human proto-oncogene in 1985 through its homology with an avian viral oncogene v-erbB and with the human EGFR (human epidermal growth factor receptor) gene. In 1987, D. Slamon with co-authors described amplification of HER2 in ~30% of clinical samples of breast cancers along with its association with aggressive disease and poor survival (1). Trastuzumab, the first HER2-targeted drug, was successfully tested in 2001 (2). Trastuzumab’s success triggered development of other HER2-targeted agents, including new antibodies (pertuzumab), kinase-inhibitors (lapatinib, neratinib), and antibody-conjugated drugs (trastuzumab-emtanasine). Numerous trials were conducted to select optimal combinations of HER2-targeting agents with cytotoxic therapies. As a result, HER2-positive breast cancer patients now have one of the best survival rates if targeted treatments are applied (3–6).

**Figure 1:**
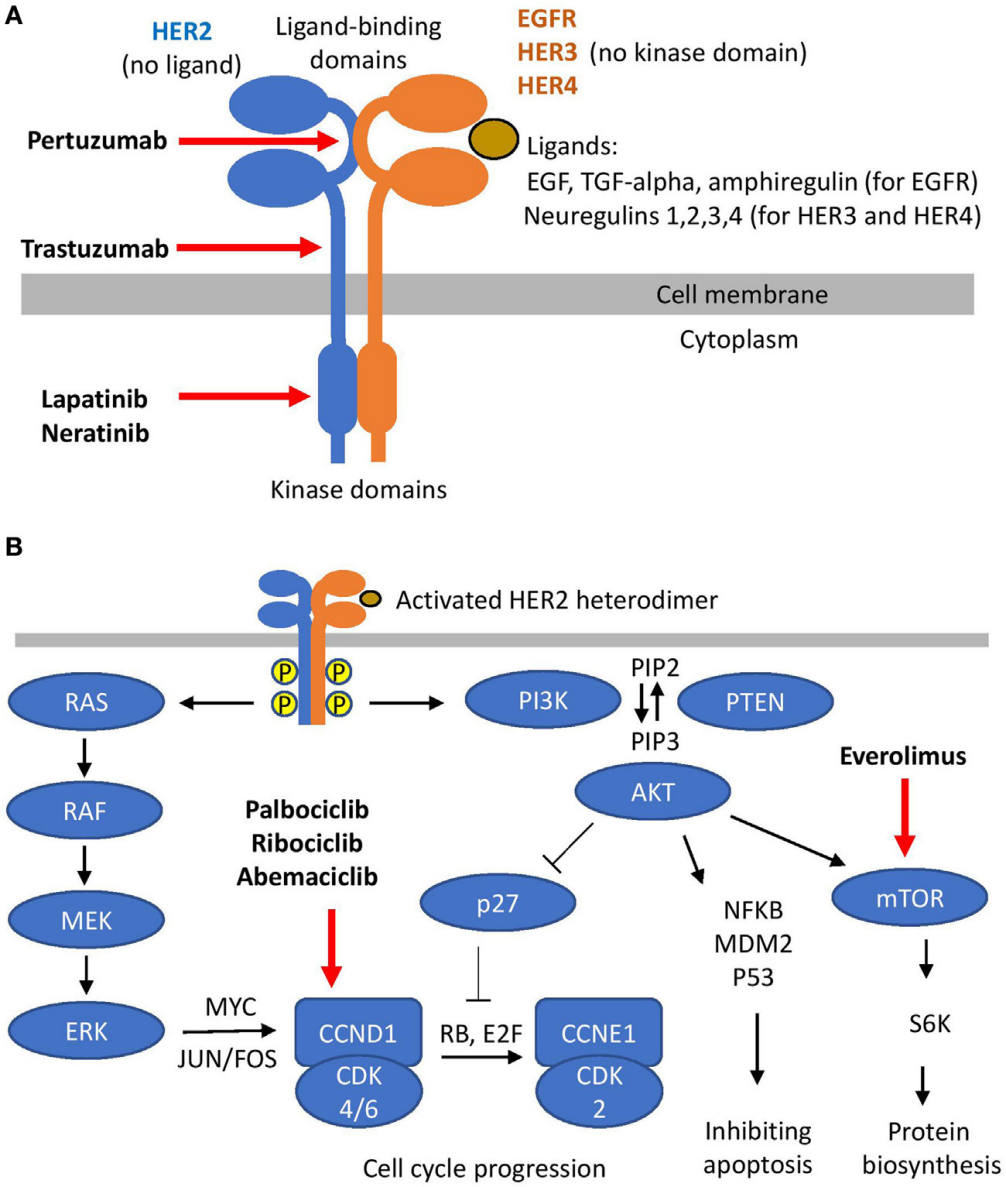
Human epidermal growth factor receptor 2 (HER2) signaling. **(A)** HER2 hetero-dimerization and HER2 targeting. **(B)** Signaling downstream of HER2 and its targeting.

Human epidermal growth factor receptor 2 is a member of human epidermal growth factor receptors (ERBB) family of membrane tyrosine-kinase receptors, which also include EGFR, HER3, and HER4 (Figure 1A). The generic structure of an ERBB receptor includes extra-cellular ligand-binding domains and an intra-cellular kinase domain. ERBB receptors are activated by a number of peptide ligands, including EGF, TGF-alpha, amphiregulin (for EGFR), and neuregulins (for HER3 and HER4). Ligand binding causes receptor dimerization, activation of the kinase domains, auto-phosphorylation, and initiation of down-stream signaling. This generic scheme has two exceptions: HER3 contains no kinase domain and HER2 has no known ligand. Thus, HER2 and HER3 rely on hetero-dimerization with other ERBB receptors to initiate their cellular effects. The key pathways downstream of HER2 include PI3K and MAPK signaling (Figure 1B) (7, 8). MAPK cascade includes sequential activation of RAS-RAF-MEK-ERK, which may stimulate proliferation through increased synthesis of CCND1 mediated by MYC and JUN/FOS transcription factors (9). The PI3K pathway starts with phosphorylation of PIP2 to PIP3, followed by activation of AKT. The de-phosphorylation of PIP3 to PIP2 is catalyzed by PTEN. The effect of PI3K-AKT signaling on proliferation may be mediated by inactivation of the p27 cell cycle inhibitor (10). Upon phosphorylation by AKT, p27 moves from the nucleus to the cytoplasm, thus allowing activation of CCNE1-CDK2 by CCND1-CDK4/6 and promoting G1-to-S cell cycle transition. Along with the effects on cell proliferation, PI3K-AKT signaling stimulates protein biosynthesis and inhibits apoptosis (11). Taken together, such downstream signaling explains the oncogenic effect of HER2 amplification in breast cancer.

## Drugs Used for Treatment of HER2-Positive Metastatic Breast Cancer

Current medical treatment of HER2-positive metastatic breast cancer (MBC) combines HER2-targeted agents with cytotoxic and hormonal therapies.

### HER2-Targeted Agents

The HER2-targeted agents currently approved for treatment of MBC, include trastuzumab, pertuzumab, lapatinib, and trastuzumab-emtansine.

#### Trastuzumab

Trastuzumab (Herceptin) is a humanized monoclonal antibody binding to the extracellular portion of HER2 close to its transmembrane domain (Figure 1A) (12). It was the first HER2-targeted agent introduced to breast cancer clinics (2) and it remains a key component of the most effective regimens used to treat HER2-positive breast cancers now (13–15). Being an anti-HER2 antibody, trastuzumab has two main mechanisms of action: (i) it suppresses HER2 signaling and (ii) it triggers an antibody-mediated immune response. Studies on cell lines allowed exploration of the effect on the signaling without interference with immune-mediated mechanisms. It was shown that trastuzumab may inhibit HER2 signaling either by destabilizing HER2 heterodimers (12) or by causing internalization of HER2 receptors with subsequent lysosomal degradation (16). In addition, trastuzumab inhibits HER2 signaling by preventing cleavage of HER2 extracellular domain (17): the cleavage of HER2 ectodomain would create a functionally active truncated isoform of HER2 (p95-HER2) (18), which contributes to the tumors progression (19). It was also suggested that trastuzumab may inhibit HER2–cSRC interaction, resulting in the activation of PTEN that attenuates PI3K-AKT signaling (20). Importantly, trastuzumab has no clinical effect on HER2-negative tumors (21). Therefore, all its clinically relevant effects are mediated through HER2.

The contribution of immune mechanisms to the trastuzumab response was confirmed by preclinical and clinical studies of Fc-gamma receptors, involved in the cell-mediated immune response. Thus, trastuzumab effect on HER2-positive xenografts depends on function of Fc-gamma receptors (22), and genetic polymorphisms of Fc-gamma receptors are associated with clinical response to trastuzumab (23). At the same time, it was noted that the contribution of immune mechanisms may be compromised by immunosuppression when trastuzumab is combined with cytotoxic agents (24).

Patent protection of Genentech’s trastuzumab (branded as Herceptin) expired in 2014 in Europe and it will expire in 2019 in the US. In 2013, a generic drug MYL-1401O with a protein sequence identical to Herceptin was manufactured by Mylan. After a phase III trial, which confirmed its clinical equivalency to Herceptin (25) in 2017, MYL-1401O (branded as Ogivri, trastuzumab-dkst) was approved by FDA as a biosimilar to Herceptin in the US. A number of other trastuzumab biosimilars have been approved in other countries.

Finally, a recent series of clinical trials demonstrated that trastuzumab can be administered subcutaneously, instead of conventional intravenous administration. This makes trastuzumab treatment more convenient for patients and reduces associated health care costs (26), especially in metastatic settings, where trastuzumab is administered to fragile patients and treatment may last for several years.

#### Pertuzumab

Pertuzumab is another monoclonal anti-HER2 antibody. It binds HER2 at a different location than trastuzumab (Figure 1A), primarily preventing formation of HER2-HER3 heterodimers (27). Pertuzumab was initially found to be effective in combination with trastuzumab (so-called “horizontal dual-blockade”) (28–30). Later, in a smaller study, pertuzumab was tested as a monotherapy; however, it showed much less efficiency than the combination (31). HER2-HER3 heterodimer is the most potent HER2 heterodimer, and combination of trastuzumab with pertuzumab is currently the most potent combination of HER2-targeted agents [as shown in CLEOPATRA trial (32) discussed in more details later].

#### Lapatinib

Lapatinib is a small-molecule tyrosine-kinase inhibitor (TKI) targeting intracellular domains of HER2 and EGFR (Figure 1A). While it showed clinical activity in HER2-positive MBC (33, 34), it is more toxic and less active than trastuzumab (35). Thus, lapatinib is reserved for use as an addition to trastuzumab (so-called “vertical dual-blockade”) (30) in later lines of treatment in patients who cannot tolerate cytotoxic chemotherapy (36, 37) or with brain metastases (as discussed in more details in Section “Targeted Treatment of Brain Metastases”) (36, 38).

#### Trastuzumab-Emtansine, T-DM1

**Figure 2:**
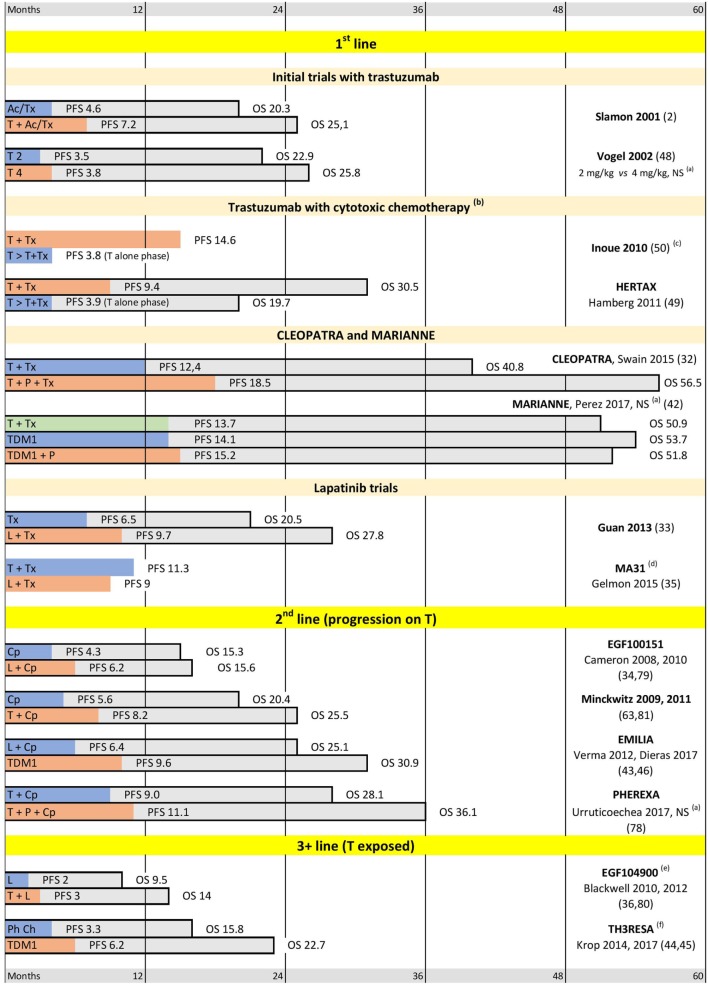
Selected trials supporting current practices in human epidermal growth factor receptor 2 (HER2)-positive metastatic breast cancer (MBC). Abbreviations: T, trastuzumab; P, pertuzumab; L, lapatinib; Tx, taxanes; Cp, capecitabine; Ac, anthracyclines; Ph Ch, physician choice. **(a)** Difference between arms is not significant. **(b)** In the sequential arm Tx is added after progression on T alone. Median progression-free survival (PFS) is shown for T alone phase only. **(c)** Median overall survival (OS) is in favor of T + Tx, median OS not reached. **(d)** OS not reported. **(e)** After 3 T-containing lines on average. **(f)** After at least 2 metastatic lines. Median PFS and OS are indicated in months.

Trastuzumab-emtansine (also known as T-DM1 or Kadcyla) is an antibody-drug conjugate (ADC). This new class of drugs allows for targeted delivery of cytotoxic molecules to the tumor, potentially increasing their efficiency and reducing their toxicity at the same time (39). T-DM1 consists of a highly potent mitotic poison maytansine (DM1) linked to trastuzumab (40). Upon binding with HER2 on tumor cells surface the drug is internalized, maytansine is released from the complex and inhibits microtubule polymerization. Tested in clinic in the 1970s, maytansine displayed an unacceptably high toxicity without the targeted delivery (41). However, trastuzumab-delivered maytansine (T-DM1) showed a good safety profile, causing fewer serious adverse effects (grade 3 or worse) than most of other treatment regimens in HER2-positive breast cancer (42–45). A series of phase-III trials showed high clinical efficacy T-DM1 in HER2-positive MBC (MARIANNE, EMILIA, and TH3RESA—see Figure 2 and Section “Key Trials With Trastuzumab-Emtansine: TH3RESA, EMILIA, and MARIANNE”). T-DM1 is superior to other current HER2-targeting therapies in the second and later lines in patients pre-treated with trastuzumab or lapatinib (45, 46). In first line treatment, T-DM1 is non-inferior to the combination of trastuzumab and taxane, while showing better tolerability (42). T-DM1 is also being tested in combination with pertuzumab and taxanes (47).

### Cytotoxic Component

Trastuzumab shows some activity as a monotherapy in HER2-positive MBC (48). However, early addition of cytotoxic chemotherapy to trastuzumab markedly improves both the response rates and the overall survival of MBC patients (see Initial Trastuzumab Trials and Figure 2: Inoue 2010 and HERTAX trials) (49, 50). These clinical data are in agreement with the preclinical models, which showed synergistic and additive interactions of trastuzumab with chemotherapeutic agents used in BC (including platinum agents, taxanes, and anthracyclines) (51). It was suggested that the synergy of trastuzumab with DNA-damaging chemotherapy, such as cross-linking platinum agents, may be explained by the inhibition of HER2-stimulated DNA-repair genes (52). Mechanism of the synergy with taxanes is less clear. However, it was hypothesized (53) that this may be mediated by inhibition of survivin expression (54, 55), which is involved in microtubule stabilization during mitosis (56).

A single cytotoxic agent is typically added to HER2-targeted treatment. Use of anthracyclines in HER2-positive MBC is limited because many patients were exposed to anthracyclines in the adjuvant setting, and because of the cumulative cardiotoxicity of anthracyclines. Importantly, the anthracyclines cardiotoxicity overlaps with the toxicity of trastuzumab leading to high rates of heart failure in this combination (57). The cardiotoxicity is substantially reduced if HER2-targeted agents are combined with taxanes instead of anthracyclines. This made taxanes the treatment of choice for the cytotoxic component in the 1st line treatment of HER2-positive MBC (e.g., see the first line trials on Figure 2) (58). The most common taxane regimens include weekly paclitaxel or docetaxel administered at 3 weeks intervals. Nab-paclitaxel may be considered for patients that do not tolerate other taxanes or steroid premedication (e.g., for diabetic patients). Vinorelbine is considered as an alternative to taxanes, since it showed similar efficacy and better tolerability in 1st line HERNATA and VELVET trials (59, 60). Attempts of adding a second cytotoxic agent, e.g., combining trastuzumab + taxanes with capecitabine (CHAT trial) or with a platinum salt (BCIRG 007 trial) improved response rates at the expense of higher toxicity. Importantly, the addition of a second cytotoxic agent has provided no survival benefit in these trials (61, 62).

There is no strong evidence to select a cytotoxic agent after taxanes. Capecitabine is a convenient oral therapy. Its combination with HER2 targeted agents showed satisfactory clinical efficiency (63–65) and it is frequently used in second line clinical trials (Figure 2). The third line cytotoxic component may include vinorelbine, if not used earlier. At later lines, the most common physician choices include gemcitabine and eribulin (45), allowing the change of cytotoxic agents along several lines of metastatic treatment.

### Hormonal Component

**Figure 3:**
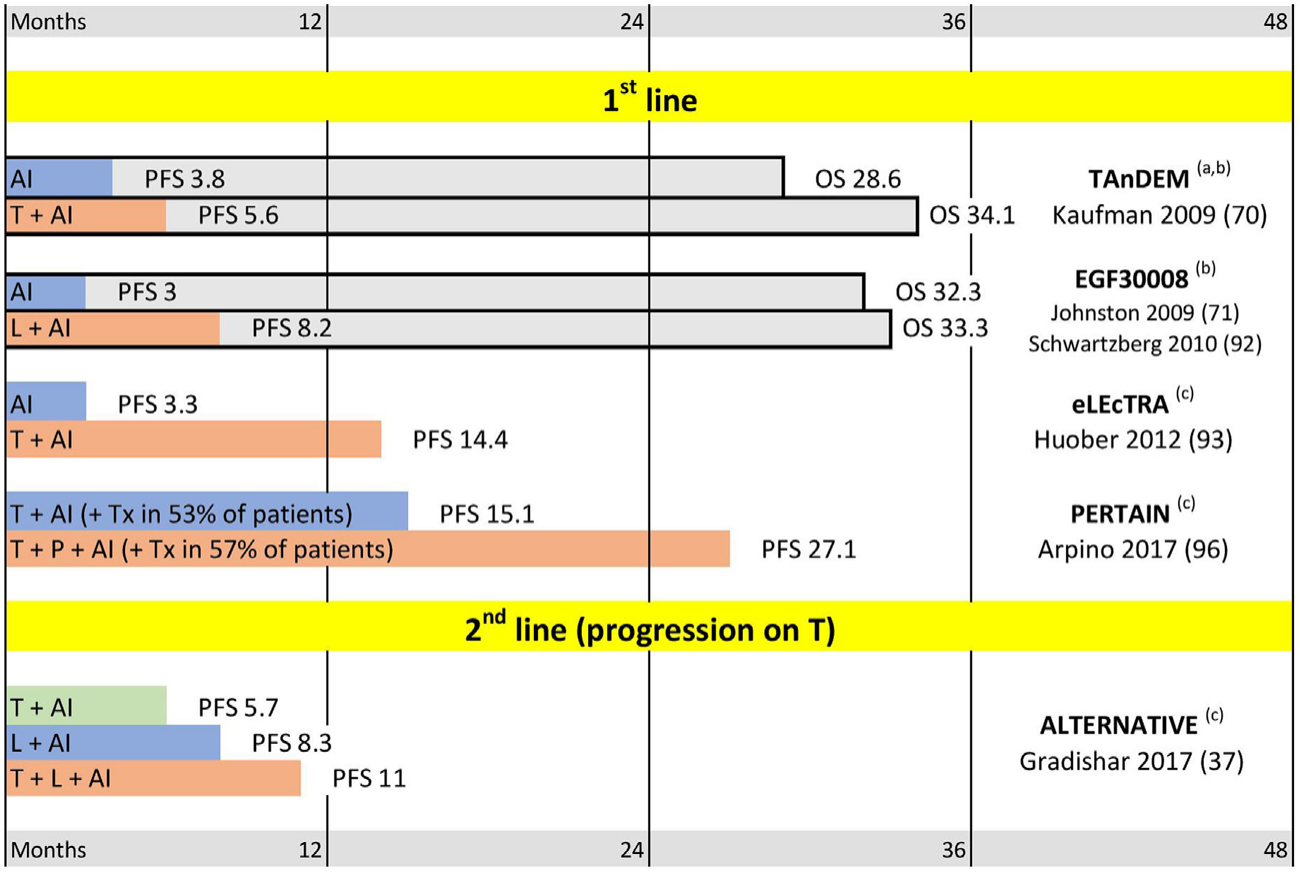
Triple positive breast cancer trials. Abbreviations: T, trastuzumab; P, pertuzumab; L, lapatinib; AI, aromatase inhibitor. **(a)** Mixed first line and later lines; median progression-free survival (PFS) and median overall survival (OS) for patients with centrally confirmed estrogen receptor. **(b)** OS trend not significant. **(c)** OS not reported. Median PFS and OS are indicated in months.

At least 50% of HER2-positive tumors express hormonal receptors (66). This group of “triple-positive” breast cancers (TPBC) has distinct biology and clinical features (67, 68). Current approaches to treatment of metastatic TPBC will be discussed in more details in Section “Treatment of Triple-Positive Breast Cancer.” However, combining HER2-targeted and cytotoxic agents with endocrine therapies is one of the obvious options for this group of patients (14, 69). It has previously been shown that the addition of HER2-targeting to endocrine treatment improves outcomes in metastatic TPBC (see TAnDEM and EGF30008 trials on Figure 3) (70, 71). At the same time, there is much less clinical evidence about the opposite comparison: addition of endocrine therapy to HER2-targeted agents in MBC, although some EBC studies suggest that this may also be beneficial (72).

Until recently, the most common endocrine agents used in BC clinics were tamoxifen (for pre-menopausal patients) and aromatase inhibitors (AIs: letrozole, anastrozole, and exemestane) for post-menopausal patients. AIs could also be considered for patients of reproductive age after ovarian ablation (e.g., with goserelin or oophorectomy). However, the preferences in selection of hormonal agents are likely to change in the view of several recent clinical trials. First, the FALCON study reported that adequately dosed fulvestrant is more effective than an AI for the first line ER-positive MBC (73). Subsequently, a series of trials showed that the addition of CDK4/6-inhibitors to endocrine treatment significantly improves PFS in metastatic setting (74–76). None of these trials have included HER2-positive patients. However, a number of studies combining HER2-targeting with fulvestrant, AIs, or CDK4/6 inhibitors are already ongoing (PATINA, PATRICIA, and monarcHER trials: NCT02947685, NCT02448420, NCT02675231).

## Key Trials Supporting Current Clinical Practice

The results of key trials supporting current clinical practice in HER2-positive MBC are summarized in Figure 2.

### Initial Trastuzumab Trials

The pivotal trial by D. Slamon (2) showed for the first time a significant improvement of both PFS and OS after addition of trastuzumab to cytotoxic chemotherapy in HER2-positive MBC. The cytotoxic component in this trial included anthracyclines or taxanes. In addition to the proof of trastuzumab efficacy, the trial also highlighted the overlapping cardiotoxicity of trastuzumab and anthracyclines (57), paving the way for the wide use of taxanes in combination with trastuzumab in metastatic settings. This also established the practice of monitoring cardiac function during trastuzumab treatment.

Effectiveness of trastuzumab as a monotherapy was demonstrated in a trial conducted by C. Vogel with co-authors (48). However, the reported PFS was noticeably inferior to the combined regimens reported earlier by D. Slamon. The direct comparison of trastuzumab mono-therapy and trastuzumab-taxane combination could be derived from the two trials reported by K. Inoue and P. Hamberg (HERTAX trial) (49, 50). These trials have a very similar design and report similar findings. For ethical reasons, it was not possible to randomly withdraw cytotoxic treatment from MBC patients. So, the comparison was designed as a randomization between simultaneous and sequential administration of cytotoxics. In the simultaneous arms, the taxanes were added to trastuzumab starting from the beginning of the treatment. In the sequential arms, the taxanes were added only after progression on trastuzumab alone. In both trials, the PFS in the trastuzumab-alone phase was markedly shorter than PFS in the trastuzumab-taxane combination. Moreover, the delay in adding cytotoxic treatment significantly reduced OS in both trials. These trials convincingly show that trastuzumab monotherapy may only be reserved for the patients who are not suitable for combined regimens.

### Dual-Block by Trastuzumab With Pertuzumab: CLEOPATRA

Further progress in treatment of HER2-positive MBC was linked to the development of “horizontal dual-blockade.” A phase III CLEOPATRA trial randomized 808 patients to either the standard trastuzumab-taxane arm or the new pertuzumab-trastuzumab-taxane combination. The addition of pertuzumab led to statistically and clinically significant improvements in both PFS and OS. In particular, the median overall survival was extended by more than a year and reached in excess of 4.5 years (32). These results are as yet unsurpassed in HER2-positive MBC (if patients are not selected by hormonal receptor level); thus, the CLEOPATRA regimen has substituted trastuzumab-taxane combination as the treatment of choice in first line metastatic treatment. At the same time, it is worth noting that only ~10% of CLEOPATRA patients were previously exposed to trastuzumab (13, 77) and a recent PHEREXA trial reported much smaller benefit from addition of pertuzumab to trastuzumab-exposed MBC patients in second line treatment (78).

### Key Trials With Trastuzumab-Emtansine: TH3RESA, EMILIA, and MARIANNE

Effectiveness of trastuzumab-emtansine (T-DM1) has been shown in a series of phase III trials, which spanned line1 (MARIANNE), line 2 (EMILIA), and later lines (TH3RESA). EMILIA and TH3RESA demonstrated superiority of T-DM1 over lapatinib + capecitabine and over physician choice in trastuzumab-exposed patients (43–46). This shifted the lapatinib + capecitabine combination to third line, while T-DM1 has been accepted as the second line treatment of choice.

The first line MARIANNE study compared T-DM1 (±pertuzumab) with trastuzumab-taxane combination (which was the first line treatment of choice at the time of the study design). T-DM1 was non-inferior to trastuzumab-taxane and showed a better safety profile (42). No trial yet makes a direct comparison between T-DM1 and CLEOPATRA protocols. It could be noted that MARIANNE enrolled more patients exposed to trastuzumab than CLEOPATRA (~30 and ~10%, respectively) (13, 77). However, in view of the clear superiority of CLEOPATRA protocol over trastuzumab-taxane, T-DM1 remains reserved for second line treatment (although there is no data about T-DM1 efficiency in pertuzumab-exposed patients).

### Selected Lapatinib Trials

Different lapatinib-containing combinations were tested in different metastatic settings. Adding lapatinib to chemotherapy significantly improves PFS and OS in first and second lines of treatment if compared to placebo (Guan-2013 and EGF00151 studies, respectively) (33, 34, 79). However, a comparison with trastuzumab showed that lapatinib-taxanes combination has lower efficacy and higher toxicity than combination of taxanes with trastuzumab (MA-31 study) (35). Similarly, despite the proven activity of the lapatinib-capecitabine protocol in the second line (EGF00151 study) (34, 79), this combination was inferior to T-DM1 in EMILIA trial (43, 46). Thus, currently lapatinib is reserved for third and later lines of treatment. Utility of lapatinib for patients with brain metastases will be discussed later in Section “Targeted Treatment of Brain Metastases.” Importantly, lapatinib is more effective in combination with trastuzumab (the “vertical dual blockade”) than taken alone, even in trastuzumab-exposed patients (see EGF104900 and ALTERNATIVE trials on Figures 2 and 3) (36, 37, 80). This is in agreement with other studies confirming efficacy of trastuzumab beyond progression (63, 81).

## Current Consensus Guidelines

Initial HER2-targeting clinical practices were shaped by trials that demonstrated effectiveness of adding trastuzumab or lapatinib to chemotherapy in HER2-positive MBC (2, 33). It was also established that HER2-targeting should continue beyond progression through multiple lines of treatment (63, 81). Thus, the general consensus at the time was that trastuzumab + taxanes could be considered as the first line treatment of choice (58, 82), followed by lapatinib- and capecitabine-containing combinations in second line (34). Overall, this practice was adopted from the time of the pivotal trial of D. Slamon in 2001 (2) until the recent development of the horizontal dual-blockade (trastuzumab + pertuzumab) and T-DM1. The first results of a large T-DM1 trial in HER2-positive MBC (EMILIA PFS results) were published in 2012 (43). Similarly, the results of TH3RESA, MARIANNE, and CLEOPATRA were published between 2014 and 2017 (32, 42, 44, 45). These trials called for a reassessment of previous clinical practices and led to updates in a number of guidelines released by expert panels around the world. The new consensus was set by the ASCO guidelines of 2014 (69), which was broadly followed by a number of other expert panels, including NCCN, ESMO, AGO and SEOM, to mention just a few within the past 3 years (13–15, 77, 83–85). The most commonly supported sequencing is summarized in Table 1. The guidelines agree that (i) dual-blockade with trastuzumab + pertuzumab is the preferred regimen for first line treatment; (ii) T-DM1 is the second line treatment of choice, and (iii) addition of lapatinib may be considered in the later lines. Pertuzumab-containing regimens and T-DM1 should also be considered in later lines for the patients, who have not received them earlier. Along with the current consensus, the guidelines highlight that the fast and simultaneous development of new HER2-targeted agents lead to a shortage of evidence regarding the use of these agents. Thus, more data is needed about the efficacy of trastuzumab-pertuzumab dual-block in trastuzumab-exposed patients. There is no data yet about efficacy of T-DM1 on pertuzumab-exposed patients or about the performance of lapatinib- and capecitabine-containing regimens on patients pre-treated with T-DM1.

**Table 1:** Current consensus sequencing of metastatic treatment in human epidermal growth factor receptor 2 (HER2)-positive metastatic breast cancer (MBC).

First line	Trastuzumab + Pertuzumab + Taxanes (vinorelbine may be considered instead of taxanes) Trastuzumab-emtansine (T-DM1) may be considered if patient is not suitable for the above or in case of a fast progression on/after adjuvant Trastuzumab

Second line	Trastuzumab-emtansine (T-DM1) Trastuzumab + pertuzumab + cytotoxic chemotherapy (taxanes, vinorelbine, or capecitabine) may be considered if not exposed to pertuzumab previously

Third line	Regimens currently recommended for first or second line should be considered for the later lines, if not used previously Trastuzumab or lapatinib + cytotoxic chemotherapy (including vinorelbine, capecitabine, gemcitabine, eribulin, and others, if not used previously) Trastuzumab + lapatinib if not suitable for cytotoxic chemotherapy

*Addition of endocrine therapy to HER2-targeted agents should be considered at any line after completion of cytotoxic chemotherapy in ER-positive patients*.

*Based on ASCO 2014 (69), NCCN 2017 (14), ESMO-ABC3 2017 (13, 77), AGO 2015 (85), and SEOM 2015-2017 (15, 83, 84) guidelines*.

## Remaining Questions

In addition to the shortage of data about novel pertuzumab and TDM1-containing regimens, there is a list of other questions, which need to be further studied to improve treatment of HER2-positive MBC. These questions include (i) use of hormonal therapy in triple-positive breast cancers, (ii) treatment of brain metastases, (iii) assessment and conversion of HER2 expression in metastatic lesions, (vi) duration of treatment for metastatic patients with stable and complete response, (v) shortage of evidence about cancers resistant to adjuvant treatment, and (vi) treatment adjustments for elderly patients. The first two questions are of special importance in this list because they immediately affect treatment for a large proportion of HER2-positive MBC patients.

### Treatment of Triple-Positive Breast Cancer

More than 50% of HER2-positive tumors also express estrogen receptors (66). These tumors are being increasingly recognized as a distinct group of breast cancers: “Triple Positive Breast Cancer” (TPBC) (68, 86, 87), whose unique biology is shaped by a complex interplay of HER2 and ER signaling (88–91). Optimal management of TPBC lies on the border between endocrine and HER2-targeted treatments. However, till recently, HER2-positive tumors have usually been excluded from endocrine trials, and studies of HER2-targeted agents often reported results without splitting them into ER-positive and ER-negative subgroups. So far, only a limited number of trials focused on metastatic TPBC patients (Figure 3). Three trials (TANDEM, EGF30008, and ELECTRA) showed that addition of HER2-targeting to endocrine treatment significantly prolongs PFS, although the OS improvements in these trials did not reach statistical significance (70, 71, 92, 93). No randomized study has yet performed the opposite comparison: the addition of endocrine treatment to HER2-targeting agents. However, a retrospective analysis suggests that this may improve the results of treatment (72). Endocrine treatment has low toxicity, and multiple lines of evidence suggest that ER-positive tumors may have inferior response to cytotoxic treatment (94, 95). Taken together, these data justify addition of endocrine treatment whenever possible for ER-positive breast cancer, which led to the current ASCO and ESMO recommendations to add endocrine agents to the treatment for most TPBC patients in metastatic setting. Addition of the hormonal agents to HER2-targeted treatment is recommended after completion of cytotoxic chemotherapy (13, 69, 77). Importantly, the guidelines emphasize that addition of endocrine therapy is not based on direct evidence. Also, they provide no reason why endocrine therapy should be delayed until completion of cytotoxic treatment.

The shortage of evidence about TPBC patients is being addressed by several recent and ongoing clinical trials summarized in Figure 3 and Table 2. The recently reported PERTAIN trial was designed to evaluate the effect of horizontal dual-blockade in metastatic TPBC (96). The control arm included trastuzumab + aromatase inhibitor, while the experimental arm added pertuzumab to the combination. Induction chemotherapy with taxanes prior the aromatase inhibitor was permitted at the discretion of the investigator (and was applied in ~50% cases in both arms). The analysis showed a significant increase in PFS after addition of pertuzumab. However, PERTAIN’s results may also be considered in a wider context. It was the first controlled trial that reported results of combining HER2-targeting, cytotoxic, and hormonal treatment at the same time—and the effect of such combination in both PERTAIN arms was unprecedented. Thus, median PFS in the experimental arm exceeded 2 years (27.1 months), which is an exceptional result for a *metastatic* cancer. Although PFS in the control PERTAIN’s arm was “just” 15.1 month, it was comparable to PFSs reported in experimental arms by MARIANNE and CLEOPATRA (14.1 and 18.5 months) despite the fact that only half of PERTAIN patients received taxanes. Another interesting observation about PERTAIN was the very fact that investigators opted not to include taxanes in nearly 50% of cases, even though it was permitted by the protocol. The treatment sub-groups analysis (for patients with and without taxanes) and overall survival data of PERTAIN trial are awaited with interest.

**Table 2:** Ongoing clinical trials in metastatic triple positive breast cancer (TPBC).

PATRICIA NCT02448420 Phase II	Postmenopausal patients exposed to trastuzumab in previous metastatic setting 3 arms: Estrogen receptor (ER) negative, human epidermal growth factor receptor 2 (HER2) positive patients: palbociclib + trastuzumab ER positive, HER2 positive patients: palbociclib + trastuzumab ER positive, HER2 positive patients: palbociclib + trastuzumab + letrozole Expected completion: 2019

monarcHER NCT02675231 Phase II	TPBC after at least two HER2-targeted therapies for advanced disease 3 arms: Abemaciclib + Trastuzumab + Fulvestrant Abemaciclib + Trastuzumab Trastuzumab + Chemotherapy of physician choice Expected completion: 2018–2019

PATINA NCT02947685 Phase III	First line metastatic TPBC patients 2 arms: Palbociclib + Anti-HER2 therapy + Endocrine therapy Anti-HER2 therapy + Endocrine therapy Induction cytotoxic chemotherapy is allowed prior randomization Expected completion: 2020–2024

DETECT-V/CHEVENDO NCT02344472 Phase III	TPBC patients with up to two prior therapies for the metastatic disease 2 arms: Trastuzumab + Pertuzumab + Cytotoxic chemotherapy Trastuzumab + Pertuzumab + Endocrine therapy Quality of life and adverse effects as a primary outcome Expected completion: 2021

SYSUCC-002 NCT01950182 Phase III	First line metastatic TPBC patients 2 arms: Trastuzumab + Cytotoxic chemotherapy Trastuzumab + Endocrine therapy Expected completion: 2018

NCT03054363 Phase Ib–II	Tucatinib in combination with Palbociclib and Letrozole for first or second line metastatic TPBC. In 2017, the study is not yet open for participant recruitment

The second recently reported metastatic TPBC trial is ALTERNATIVE (37). The study enrolled TPBC patients progressing on a (neo)adjuvant/first line trastuzumab + chemotherapy regimen. The treatment included three arms: trastuzumab vs. lapatinib vs. trastuzumab + lapatinib. Aromatase inhibitors were added in each arm. Patients intended for cytotoxic chemotherapy were excluded from this trial. The vertical dual blockade (trastuzumab + lapatinib combination) showed superior PFS as compared with lapatinib or trastuzumab alone (11, 8.3, and 5.7 months, respectively). Again, despite the absence of cytotoxic treatment in this trial, the 11 months median PFS achieved in trastuzumab + lapatinib + AI arm was among the best reported in HER2-positive second line trials, such as EMILIA (9.6 months PFS for T-DM1) or PHEREXA (11.1 months for horizontal dual-block + taxanes) (43, 78). It is not yet clear whether the exceptional PFS results reported by PERTAIN and ALTERNATIVE reflect the favorable intrinsic properties of TPBC or the good response to combined HER2- and ER-targeting; and these trials have not yet reported the overall survival data. However, these trials already provide interesting and encouraging data about new treatments and outcomes in metastatic TPBC.

A significant progress has been made over the recent years in the treatment of metastatic ER-positive HER2-negative breast cancer: fulvestrant (500 mg) has been promoted to first line metastatic hormonal treatment (73) and CDK4/6 inhibitors have been established as a standard addition to endocrine agents (97). This progress triggered a series of trials testing whether these new approaches could be beneficial for TPBC patients (Table 2). Thus, phase II PATRICIA and monarcHER trials are studying addition of CDK4/6 inhibitors to trastuzumab (with or without hormonal treatment) in patients progressing after previous trastuzumab-containing metastatic regimens. Phase III PATINA trial studies addition of CDK4/6 inhibitors to HER2-targeted agents in first line metastatic treatment (with or without hormonal therapy). Two other ongoing trials compare endocrine and cytotoxic therapies in combination with HER2 targeting in metastatic TPBC (DETECT-V and SYSUCC-002 trials, see details in Table 2). All these trials are currently enrolling patients. The results are expected between 2018 and 2024.

Taken together, the recently reported and ongoing trials in metastatic TPBC will provide evidence to support new treatment standards for this group of patients. Finally, it could be noted that most metastatic TPBC patients were exposed to previous endocrine treatments in a (neo)adjuvant setting and/or in previous metastatic lines. Thus, testing for activating mutations in ligand-binding domain of estrogen receptor may be helpful for rational selection of the endocrine component of their treatment (98).

### Targeted Treatment of Brain Metastases

Brain metastases (BM) have a distinct biology and poor prognosis (99, 100). Currently, symptomatic BM is reported in 20–40% of HER2-positive MBC (101, 102). Additionally, up to 10% of patients may have asymptomatic BM detectable on autopsy (100). Incidence of BM is significantly higher in ER-negative than in ER-positive disease (99). Treatment of BM is based on radiotherapy and surgery, supported by systemic therapies based on cytotoxic and targeted agents (103–105). The backbone systemic therapy could be combined with additional medication (such as corticosteroids and anti-epileptic drugs) that is beyond the scope of this review (106). Paradoxically, treatment with trastuzumab increases incidence of brain metastases (107–110), while prolonging the time to BM (110) and overall survival of patients with BM (108, 111). This is commonly explained by trastuzumab’s success in suppressing extracranial metastatic sites, which allows time for brain metastases to develop and manifest clinically. Importantly, while achieving good extra-cranial control, trastuzumab has limited effect on brain metastases themselves because of its poor permeability through the blood–brain barrier (BBB). Thus, under conditions of unimpaired BBB, trastuzumab’s concentration in cerebrospinal fluid could be 420 times below its concentration in serum; even when BBB is compromised by irradiation, the concentration of trastuzumab in brain is still about 50 times lower than in blood (112). In contrast, small-molecule TKIs, like lapatinib, penetrate BBB much better (113). This raised expectations about a possible special role for TKIs in BM treatment and encouraged a number of small trials evaluating effect of lapatinib on BM in heavily pre-treated HER2-positive MBC patients (114). In these trials, lapatinib alone showed low CNS response rates (2–6%) and low PFS (up to 3 months). However, when lapatinib was combined with capecitabine, the objective response rates rose to 18–38% and PFS to ~5 months. For comparison, CNS PFS reported for cytotoxic chemotherapies alone did not exceed 4 months at the time (114). Taken together, these trials suggested some CNS benefit from addition of lapatinib, although of a limited clinical significance in pre-treated population. In 2013, a small single-arm open-label trial tested lapatinib + capecitabine combination on patients with previously untreated brain metastases (LANDSCAPE trial). It reported 66% CNS ORR and median PFS of 5.5 months (115). Excellent ORR in LANDSCAPE trial was reflected in some guidelines, including ASCO-2014 and EANO-2017 (104, 105). However, a later CEREBEL trial failed to show superiority of lapatinib + capecitabine over trastuzumab in preventing BM (38). Although interpretation of the CEREBEL trial is complicated by the unexpectedly low rates of BM (3–5%), this trial brought some caution toward the lapatinib + capecitabine regimen. More importantly, a sub-analysis of EMILIA trial directly showed that T-DM1 provides better OS than lapatinib + capecitabine in patients with BM (116). Similarly, trastuzumab and/or pertuzumab trials showed improvement in overall survival for patients with BM (111, 117), despite the low penetrance of BBB for these drugs. This is commonly explained by the importance of extracranial control in patients with BM. Building on this evidence, the most recent ABC-3 guideline from ESO-ESMO does not recommend a change of systemic treatment for patients with BM, providing stable extracranial disease (13, 77). A trastuzumab-containing regimen is still essential for patients with BM because of its contribution to extracranial control. However, considering the low penetrance of BBB for trastuzumab, patients with BM may be encouraged to participate in clinical trials (85, 105). Trials of new TKIs, such as neratinib, are underway (118). Future trials combining small molecules and antibody-based agents may also be expected, as well as the trials of new methods to increase BBB permeability (119).

## Therapies Under Development

The HER2-targeted treatments developed over the past 20 years converted the unfavorable natural history of HER2-positive BC into favorable clinical outcomes. At the same time, the metastatic disease still remains incurable for most patients. The innate and acquired resistance to existing therapies requires development of novel agents and strategies. The range of new therapies that are currently under development for HER2-positive MBC include (i) new TKIs targeting ERBB receptors and inhibitors of their down-stream signaling, (ii) inhibitors of cell cycle, HSP90, and angiogenesis, (iii) HER2-targeted drug delivery, and (iv) new antibodies and immunotherapy aimed at HER2-positive BC.

### New ERBB-Targeting TKIs

Neratinib is classified as a pan-ERBB TKI because it binds and irreversibly inhibits all ERBB receptors except HER3 (118, 120). On the basis of ExteNET study, neratinib has been recently approved by the FDA for extended post-trastuzumab adjuvant treatment (121). However, neratinib yet failed to show superiority over comparators in metastatic settings. Comparison of neratininb with trastuzumab (both in combination with taxanes) in first line treatment was performed by NEfERT trial, which reported identical PFS in both arms (12.9 months) and much higher toxicity in the neratinib arm (grade 3 diarrhoea developed in up to 30% of patients) (122). In a second line trial that compared neratinib monotherapy with the combination of lapatinib + capecitabine, the neratinib arm showed shorter PFS and OS than the combination (PFS 4.5 vs 6.8 and OS 19.7 vs 23.6 months, respectively, differences between arms are not significant) (123). At the same time, a sub-analysis of NEfERT-T trial showed that neratinib was more effective against brain metastases (relative risk of CNS recurrences 0.48, *p* = 0.002). Another pan-ERBB TKI, afatinib, was tested in several breast cancer trials, and has progressed to a phase III trial in the first line metastatic setting (LUX-Breast 1). However, this trial was stopped after an interim assessment showed that afatinib outcomes were less favorable than trastuzumab (124). Neither neratinib nor afatinib are currently approved for treatment of HER2-positive MBC outside of clinical trials (125), although new trials of neratinib may be expected in patients with brain metastases.

As an alternative to pan-ERBB inhibitors, a selective HER2-specific TKI was developed by Array BioPharma (Tucatinib, formerly known as ONT-380 or ARRY-380). It was expected to reduce the off-target effects and high toxicity common for dual- and pan-ERBB inhibitors. In a phase I trial, Tucatinib showed promising efficacy and safety profile (126). It also showed capacity to penetrate the BBB and some promising results in patients with brain metastases (127). In view of these phase I results, a phase II trial has been recently initiated, which combines tucatinib with trastuzumab and capecitabine in HER2-positive MBC (HER2CLIMB, NCT02614794). Another recently initialed tucatinib trial combines it with CDK4/6 and aromatase inhibitors in metastatic TPBC patients (NCT03054363). The results of these trials are awaited with interest.

### PI3K and mTOR Inhibitors

PI3K and mTOR are key components in signaling downstream of HER2 (Figure 1B). The m-TOR inhibitor everolimus has been extensively studied in breast cancer, and it is approved for treatment of ER-positive HER2-negative BC on the basis of BOLERO-2 trial (128). However, it showed less encouraging results in two large phase III trials in HER2-positive MBC (BOLERO-1 and BOLERO-3). BOLERO-1, which studied addition of everolimus to trastuzumab + taxanes in first line metastatic treatment, reported that everolimus was associated with increased toxicity without any improvement in either PFS or OS (129, 130). BOLERO-3 tested addition of everolimus to trastuzumab + vinorelbine in second line metastatic treatment for patients previously exposed to trastuzumab and taxanes. The addition of everolimus in the BOLERO-3 led to a statistically significant improvement of PFS (131). However, numerically the median PFS was prolonged by less than 2 months (7.0 vs 5.8 months) and there was no improvement in OS (132). A joint analysis of BOLERO-1 and BOLERO-3 was conducted in search for biomarkers to select patients benefiting most from treatment with everolimus. It showed that somatic mutations in the PI3K pathway (PIK3CA, PTEN, and AKT1 genes) could be used to predict response, and that everolimus benefit was mainly limited to ER-negative patients (133).

Similarly, several PI3K inhibitors which have been previously studied in ER-positive HER2-negative BC, are currently at early stages of testing in HER2-positive disease (including buparlisib (134), alpelisib (135), taselisib-NCT02390427, and pictilisib-NCT00960960). Like everolimus, none of these PI3K inhibitors is yet recommended for HER2-positive patients outside of clinical trials. However, they may be used in future personalized treatment of HER2-positive MBC if companion biomarkers are developed to select responsive patients.

### CDK4/6 Inhibitors

CDK4 and CDK6 are partners of Cyclin D1, the key cell cycle regulator controlling G1-S transition. Activated by Cyclin D1, CDKs 4/6 phosphorylate RB protein, which in turn releases E2F from RB-E2F complexes leading to activation of Cyclin E and downstream cell cycle machinery (Figure 1B) (136). Cyclin D1 is frequently amplified in human cancers, including breast cancer (137). Surprisingly, despite the central role of Cyclin D1 in cell cycle regulation, mice lacking Cyclin D1 are viable and resistant to HER2(neu)-induced breast cancers (but not to breast cancers induced by MYC) (138). HER2-amlpified breast cancer cell lines are among the most sensitive to CDK4/6 inhibition (74). Along with the involvement in HER2-dependent carcinogenesis, Cyclin D1 also plays a key role in signaling downstream of estrogen receptor (139). These molecular findings triggered interest in CDK4/6 inhibitors for breast cancer treatment, culminating in several recently published large phase III clinical trials (PALOMA-2/3, MONALEESA-2, and MONARCH-2) that tested addition of different CDK4/6 inhibitors (palbociclib, ribociclib, and abemaciclib, respectively) to hormonal therapies in ER-positive HER2-negative MBC (75, 97, 140, 141). The success of these trials led to regulatory approvals and supported a wave of interest in CDK4/6 inhibitors for treatment of HER2-positive disease. Several ongoing CDK4/6 trials in HER2-positive MBC were discussed in the TPBC section of this review (PATRICIA, monarcHER, and PATINA, see Table 2). Notably, one of the PATRICIA arms also includes ER-negative patients. It was also reported that abemaciclib has good CNS penetration, showing similar concentrations in plasma and brain (142). Importantly, the effect of CDK4/6 inhibitors requires intact RB protein expression (143). Thus, RB loss (and other RB mutations) should be monitored before treatment with CDK4/6 inhibitors and after progression on treatment. At present, CDK4/6 inhibition is perceived as one of the most promising new directions in the treatment of HER2-positive MBC, which may soon be used in clinical practice (at least in TPBC patients) (144).

### Inhibitors of HSP90 and Angiogenesis

Experimental studies suggested a potential role for HSP90, angiogenesis, and topoisomerase inhibitors in treatment of different types of cancer. However, clinical trials have not yet justified including these drugs into routine clinical practice in HER2-positive MBC.

HSP90 is a chaperon protein, which is required for proper folding and function of hundreds of its client proteins (145), including HER2 and its key downstream targets (146). Preclinical studies reported anti-tumor activity of HSP90 inhibitors on a wide range of BC cells, with HER2-positive cell lines being particularly sensitive (147). In 2011, a phase II clinical trial studied effect of an HSP90 inhibitor tanespimycin on 31 HER2-positive MBC patients who previously progressed on trastuzumab (148). It reported objective responses to the tanespimycin + trastuzumab combination in 22% of the patients with median PFS of 6 months and median OS of 17 months. While this was a positive result, the efficiency of tanespimycin + trastuzumab looked modest in comparison to trastuzumab-emtansine reported shortly afterward (EMILIA trial first reported in 2012: PFS 9.6 months and OS 30.9 months) (43). Another HSP90 inhibitor showed limited efficacy in HER2-positive MBC in a phase II trial reported in 2013 (149). No further clinical trials of HSP90 inhibitors were reported in HER2-positive MBC since then. However, the overall interest and experimental studies of HSP90 are continuing, with focus on biological complexity of HSP90 functions in cancer (145, 150, 151).

Similarly, there were attempts to inhibit angiogenesis in HER2-positive metastatic cancer. Angiogenesis is highly specific for tumor tissue in adults (152). HER2 overexpression stimulates VEGF expression in breast cancer (153), potentially leading to poorer clinical outcomes (154). Phase I and II trials showed that inhibitors of angiogenesis (such as VEGF inhibitor bevacizumab) may have effect on some HER2-positive MBC (155). However, a phase III clinical trial of bevacizumab in HER2-positive MBC failed to detect an improvement in PFS in first line treatment (AVEREL trial) (156), which was consistent with an unsuccessful adjuvant trial in HER-positive EBC (BETH trial) (157). Since 2010, the FDA does not approve bevacizumab for any form of breast cancer. The current studies of angiogenesis inhibitors in breast tumors are focused on the search for a sub-set of tumors that may be responsive to this class of drugs (158, 159).

### HER2-Targeted Drug Delivery and Immunotherapies

The success of T-DM1 stimulated development of other HER2-targeted antibody-drug-conjugates (ADC). The main directions in development of new ADC include (i) new targeting antibodies, (ii) new conjugated drugs, and (iii) increasing the drug-to-antibody ratio. Thus, DS-8201 is a conjugate of trastuzumab with a topoisomerase-1 inhibitor (160). Its development progressed to a phase I clinical trial, which confirmed safety and potential efficacy in T-DM1-exposed patients (161). Another novel ADC is XMT-1522. Its antibody part targets a new epitope on HER2, thus avoiding potential competition for epitopes with trastuzumab or pertuzumab if used in combination. Moreover, each XMT-1522 antibody is conjugated with 15 molecules of a proprietary cytotoxic component auristatin. XMT-1522 showed significantly higher potency than T-DM1 in preclinical models (162). Currently, it is being tested in a phase I clinical trial (NCT02952729).

An alternative approach to HER-2 targeted drug delivery was tested by an antibody-conjugated liposomal doxorubicin formulation called MM-302. It delivered drug to tumor in liposomes tagged with anti-HER2 antibodies. The intention was to expand doxorubicin therapeutic range by avoiding its cardiotoxicity. A recent phase II trial (HERMIONE) compared the combination of trastuzumab with MM-302 against a combination of trastuzumab with cytotoxic chemotherapy of physician choice in heavily pre-treated HER2-positive MBC patients (163). MM-302 was well tolerated. However, the trial has been terminated after an interim review showed no efficacy benefit over the comparator. Another new strategy is non-antibody targeting. Thus, S. Pallerla with co-authors designed a HER2-targeted doxorubicin-peptidomimetic conjugate (164). The targeting is achieved by a short peptide that selectively binds to HER2. The compound is still in an early preclinical phase. An interesting approach was reported by X. Kang with co-authors, who conjugated trastuzumab with gold nanoparticles, which allowed HER2-targeted photothermal tumor destruction (165). Potentially, this method avoids any chemotherapy-associated toxicity. However, the studies are also still at a preclinical phase (testing on xenografts).

Finally, it is well established that immune mechanisms may contribute to response to trastuzumab (see Trastuzumab Section) (22, 23). A new antibody, magretuximab (MGAH22), has been designed to enhance the antibody-dependent cell-mediated cytotoxicity against HER2-positive tumor cells. Its Fab region targets the same epitope as trastuzumab. However, its Fc region was modified to increase affinity to activating Fc-receptors on NK-cells and macrophages. Magretuximab was well tolerated and showed promising efficacy as a monotherapy in phase I clinical trial (166). A phase III trial, SOPHIA, is testing a combination of magretuximab + cytotoxic chemotherapy in trastuzumab-exposed MBC patients (167). An alternative approach is used in ertumaxomab: an engineered multi-functional antibody that simultaneously binds HER2, CD3, and the Fc-gamma-receptors. Potentially, this might promote immune response by bringing the tumor cell, T-cell, and accessory immune cells together. So far, ertumaxomab has been tested in two phase I clinical trials that reported encouraging safety and efficacy (168, 169). Other immunotherapy approaches that already reached at least phase I–II trials, include immune checkpoints inhibitors (PANACEA and KATE2 trials: NCT02129556, NCT02924883), T-cell therapies (170), and vaccines (171). Overall, despite still being at early stages of development, the new HER2-targeted drug delivery systems and immunotherapies show promise and may extend our armamentarium for treating HER2-positive MBC in future.

## Conclusion

High expression or amplification of HER2 defines a distinct group of breast cancers. 20 years ago it was considered as an intrinsically aggressive subtype associated with poor prognosis (1). However, the tumor dependence on HER2 has also provided a target for treatments, which dramatically changed the outcomes for this type of disease. Now, HER2-positive breast cancer is considered as a favorable subtype (3–6) with about 70% of EBC patients reaching 10-year *disease-free* survival after the current HER2-targeted adjuvant treatments (172). While the metastatic HER2-positive BC remains incurable, HER2-targeting significantly prolonged the life of these patients too, with median overall survival exceeding 4 years in recent clinical trials (32, 42). HER2-targeted agents currently approved for metastatic breast cancer, include trastuzumab, pertuzumab, trastuzumab-emtansine, and lapatinib. They are used in different combinations with each other and with other chemotherapeutic agents. The recent progress in treatment of HER2-positive MBC was achieved by the development of horizontal dual blockade (pertuzumab + trastuzumab) (32) and by HER2-targeted drug delivery (trastuzumab-emtansine) (42). The current studies focus on (i) accumulating new evidence about recently established treatment regimens, (ii) treatment stratification by expression of estrogen receptors, (iii) treatment of brain metastases, (iv) development of new tools for HER2-targeting (new TKIs, antibodies, antibody-drug conjugates, and immunotherapies), and on (v) targeting pathways downstream of HER2 (including PI3K, mTOR and CDK4/6 inhibitors). Future therapies will be based on molecular profiling and companion diagnostics to allow monitoring and rational selection of targeted treatments for individual patients.

## Author Contributions

AL confirms that he wrote the review by himself in all entirety.

## Conflict of Interest Statement

The author declares that the research was conducted in the absence of any commercial or financial relationships that could be construed as a potential conflict of interest.

## Abbreviations

HER2 (1,3,4), human epidermal growth factor receptor 2 (1,3,4); EGFR, human epidermal growth factor receptor (same as HER1); ERBB, human epidermal growth factor receptors family (same as HER family; historically named after erbB gene of avian erythroblastosis virus); BC, breast cancer; MBC, metastatic breast cancer; EBC, early breast cancer; TPBC, triple positive breast cancer; PFS, median progression-free survival; OS, median overall survival; ORR, objective response rate; BM, brain metastases; BBB, blood brain barrier; AI, aromatase inhibitor; ER, estrogen receptor; NK, natural killer cells; ADC, antibody-drug conjugate; TKI, tyrosine kinase inhibitor.
